# Supplementary material for: Cargo sorting zones in the trans-Golgi network visualized by super-resolution confocal live imaging microscopy in plants
Source: Nat Commun. 2021 Mar 26;12:1901. doi: 10.1038/s41467-021-22267-0 (PMC7997971; doi:10.1038/s41467-021-22267-0)
Supplement: Supplementary file 1 — Supplementary Information [file 41467_2021_22267_MOESM1_ESM.pdf]

Supplementary Information

Cargo sorting zones in the *trans*-Golgi network visualized by super-resolution confocal live imaging microscopy in plants

Yutaro Shimizu, Junpei Takagi, Emi Ito, Yoko Ito, Kazuo Ebine, Yamato Komatsu, Yumi Goto, Mayuko Sato, Kiminori Toyooka, Takashi Ueda, Kazuo Kurokawa, *Tomohiro Uemura, and *Akihiko Nakano.

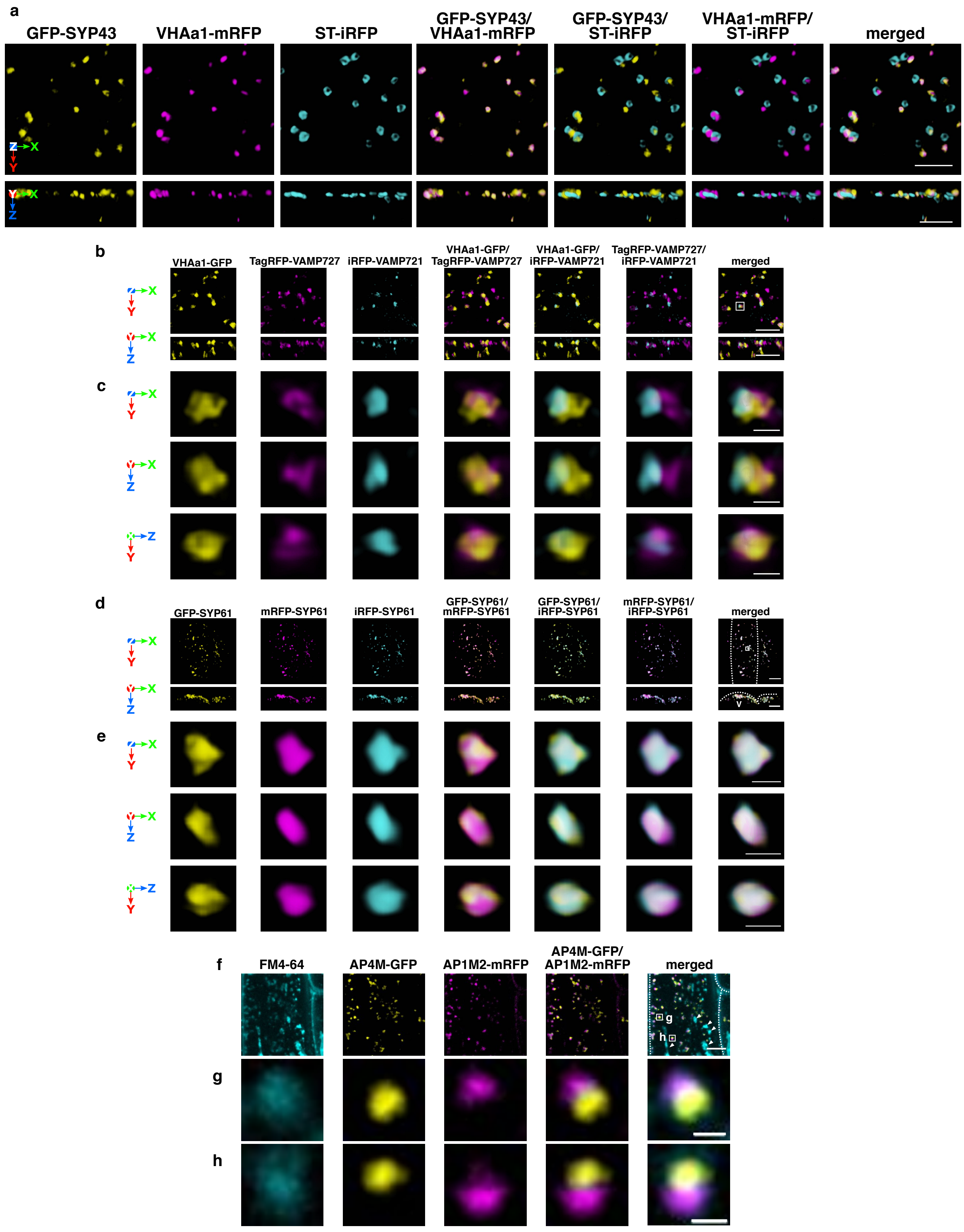

Supplementary Fig. 1 Triple-color imaging of the TGN.

a–d 3-color SCLIM imaging of root epidermal cells in the elongation zone of Arabidopsis expressing GFP-SYP43 × VHAa1-mRFP × ST-iRFP **(a)**, Arabidopsis expressing VHAa1-GFP × TagRFP-VAMP727 × iRFP-VAMP721 **(b, c)**, or GFP-SYP61 × mRFP-SYP61× iRFP-SYP61 **(d, e)**. **a, b, d** 3D images. **c, e** Multi-angle magnified 3D images of the boxed area in **a** and **c,** respectively.

f–h Confocal images of FM4-64 × AP4M-GFP × AP1M2-mRFP under conventional CLSM with a high-resolution objective lens after 6 min of FM4-64 uptake. **f** Low-magnification images. **g, h** High-magnification images of the boxed area in **f**. Dashed lines indicate cell edges. Arrowheads show strong fluorescence signals of FM4-64 from the plasma membrane/ cell wall. V, vacuole area. Scale bars = 5 µm **(a, b, d, f)**; 1 µm **(c, e, g, h)**. The experiments were repeated two times **(a–c, f–h)** and five times **(d, e)** with similar results, and micrographs from representative experiments are presented.

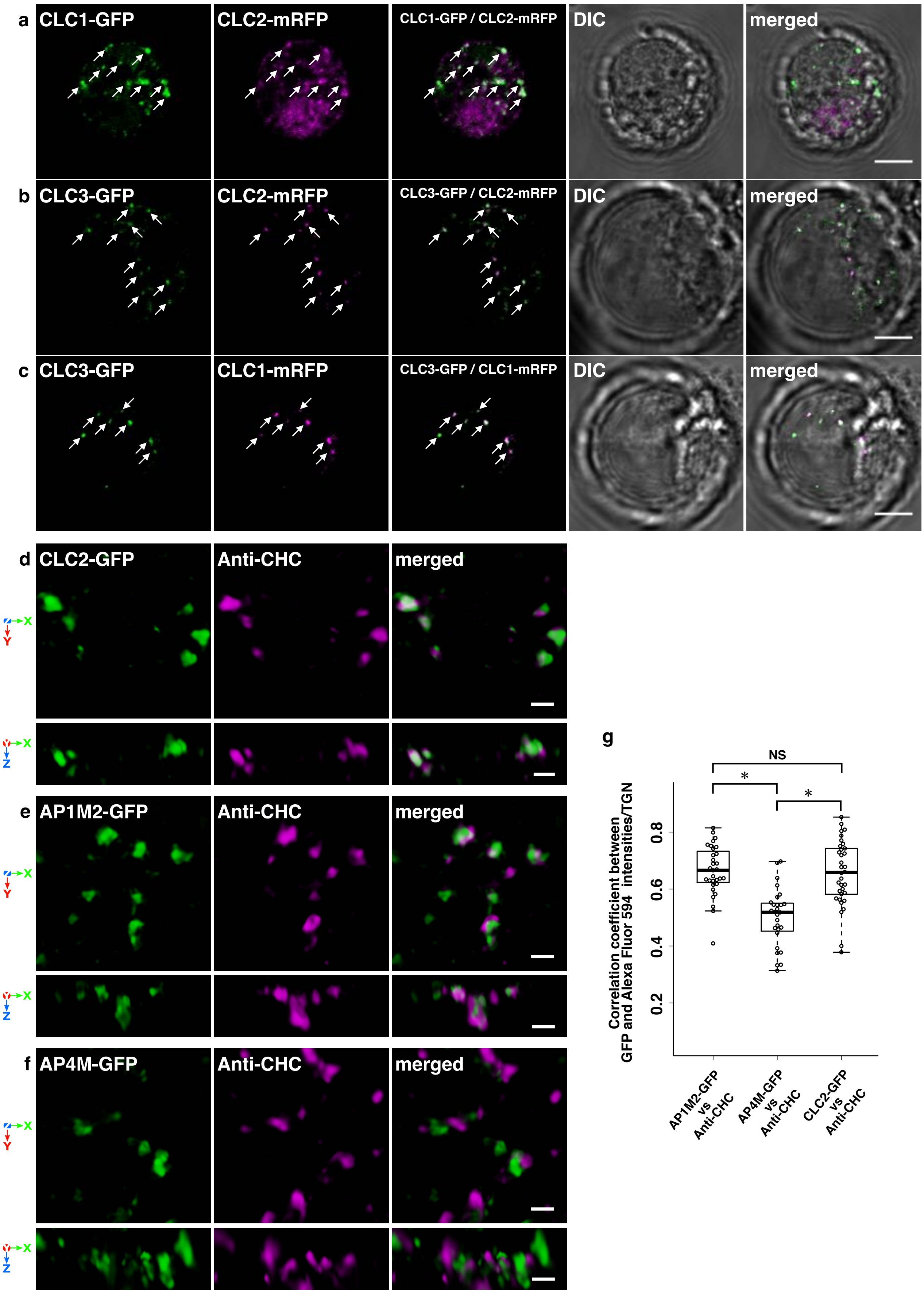

Supplementary Fig. 2 Subcellular localization of fluorescent protein-tagged CLCs or endogenous CHCs.

a–c Confocal images of CLC1-GFP and CLC2-mRFP **(a)**, CLC3-GFP and CLC2-mRFP **(b)**, or CLC3-GFP and CLC1-mRFP **(c)** transiently expressed in the protoplasts of Arabidopsis suspension cultured cells.
d–g 3D images of CLC2-GFP, AP1M2-GFP, and AP4M-GFP with anti-CHCs (Alexa 594) **(d, e, and f, respectively)** in the epidermal cells of the root elongation zone under SCLIM. **g** 3D colocalization analysis: n = 31, 28, and 34 TGNs for AP1M2-GFP vs Anti-CHC, AP4M-GFP vs Anti-CHC, and CLC2-GFP vs Anti-CHC, respectively, from 3 biological replicates. Two-sided Steel-Dwass test; $P = 8.3 \times 10^{-7}$ (Left: AP1M2-GFP × Anti-CHC versus AP4M-GFP × Anti-CHC), P = 0.9 (Top: AP1M2-GFP × Anti-CHC versus CLC2-GFP × Anti-CHC), and $P = 3.5 \times 10^{-6}$ (Right: AP4M-GFP × Anti-CHC versus CLC2-GFP × Anti-CHC); $^*P < 0.01$, NS = nonsignificant. Boxes represent 25% and 75% quartiles, lines within the box represent the median, and whiskers represent the minimum and maximum values within 1.5x the interquartile range. Arrows indicate colocalized signals in endomembranes. Scale bars = 5 µm **(a–c)**; 1 µm **(d–f)**. The experiments were repeated three times with similar results, and micrographs from representative experiments are presented.

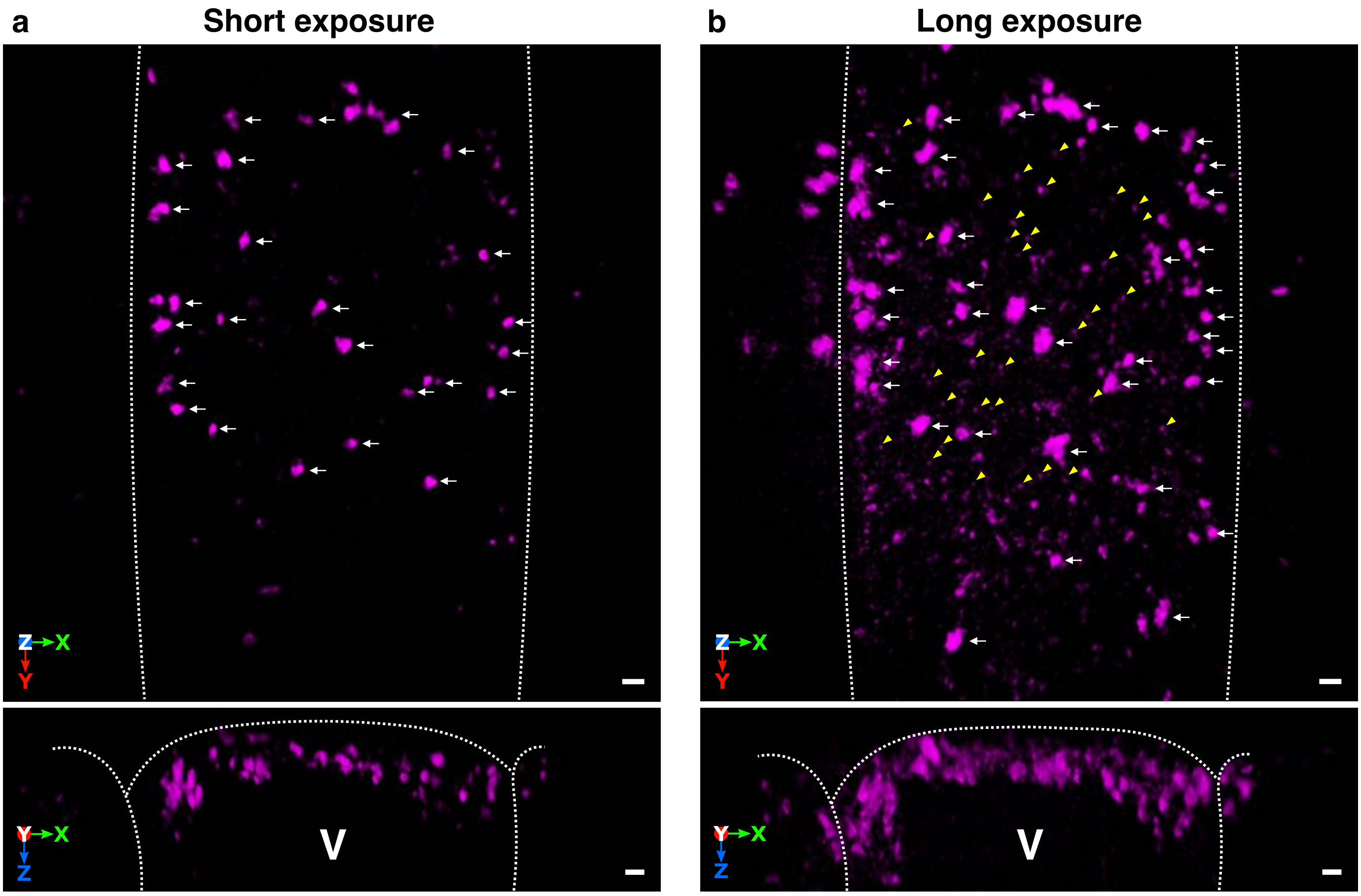

Supplementary Fig. 3 Fluorescent protein-tagged clathrin signal on the plasma membrane.

a, b Short exposure **(a)** and long exposure **(b)** 3D images of CLC2-mKO in the epidermal cell of the root elongation zone under SCLIM. Arrowheads indicate representative CLC2-mKO signals on the plasma membrane. Arrows indicate TGN-localized CLC2-mKO. Dashed lines show cell edges. V, vacuole area. Scale bars = 1µm. The experiments were repeated five times with similar results, and micrographs from representative experiments are presented.

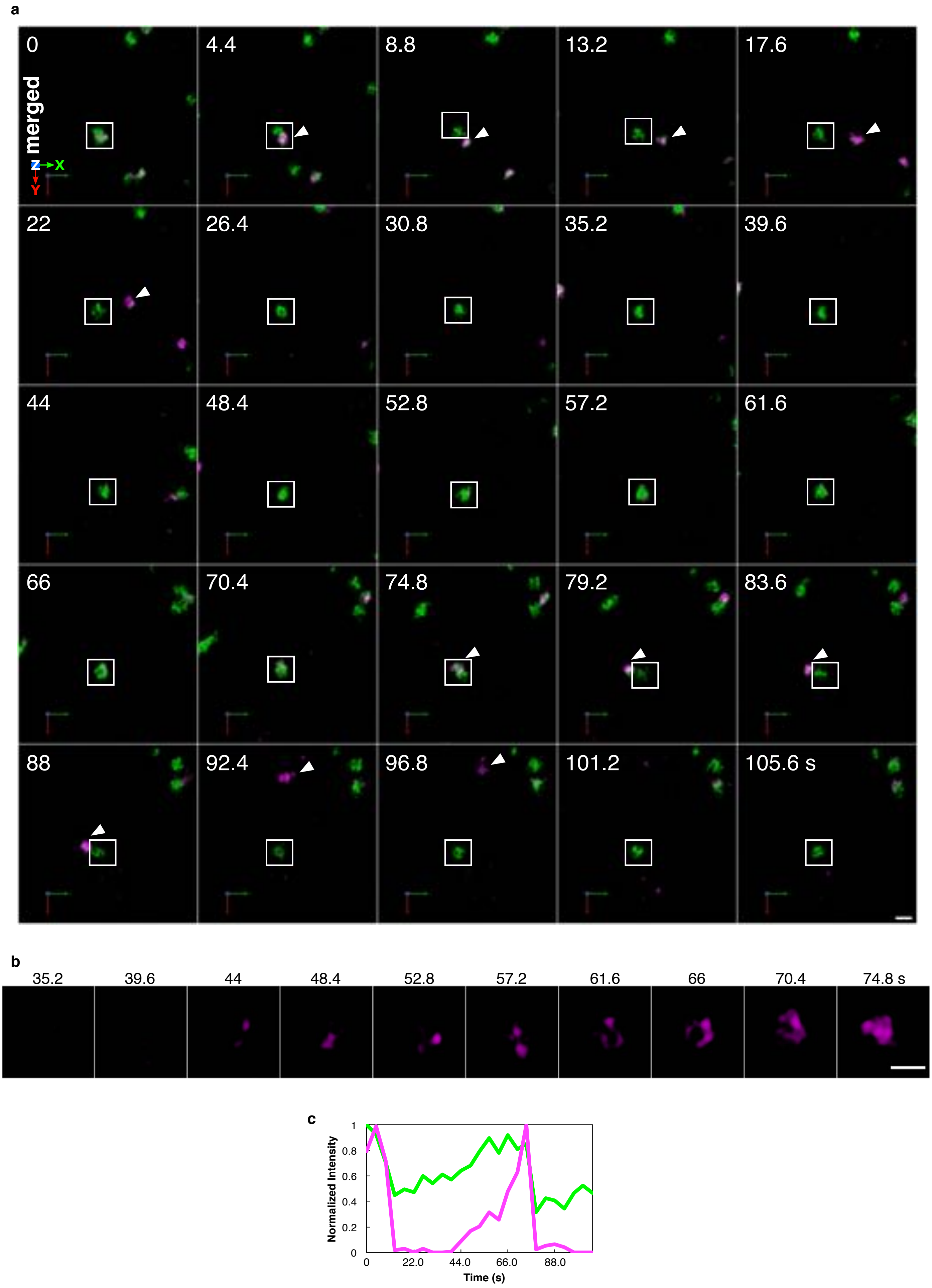

Supplementary Fig. 4 Accumulation and budding of clathrin in the TGN.

a 4D images of GFP-SYP61 and CLC2-mKO in the epidermal cell of the root elongation zone under SCLIM. Arrowheads indicate dissociation of the GI-TGN labeled with GFP-SYP61 and CLC2-mKO from the GA-TGN. **b** Magnified images of CLC2-mKO accumulation on the TGN shown in **a** (35.2 s – 74.8 s). Images are lined up every 4.4 s from left to right. Scale bars = 1 µm. **c** Temporal fluorescence profile of boxed area in **a**. The experiment was repeated three times with similar results, and micrographs from representative experiments are presented.
