# Supplementary material for: Cargo sorting zones in the trans-Golgi network visualized by super-resolution confocal live imaging microscopy in plants
Source: Nat Commun. 2021 Mar 26;12:1901. doi: 10.1038/s41467-021-22267-0 (PMC7997971; doi:10.1038/s41467-021-22267-0)
Supplement: Supplementary file 3 — Descriptions of Additional Supplementary Files [file 41467_2021_22267_MOESM3_ESM.docx]

Descriptions of Additional Supplementary Files

**Supplementary Movie 1
Description:** A rotating 3D projection image of the TGN with iRFP-SYP61 (Cyan), AP1M2-mRFP (Magenta), and AP4MGFP (Yellow) observed by SCLIM. Grid width = 0.34 µm. See also Fig. 2i–k.

**Supplementary Movie 2
Description:** 4D movie of iRFP-SYP61 (Cyan), AP1M2-mRFP (Magenta), and AP4M-GFP (Yellow) in the epidermal cell of the root elongation zone under SCLIM. Scale bar = 1µm. See also Fig. 4a.

**Supplementary Movie 3
Description:** 4D movie of AP1M2-GFP (Green) and CLC2-mKO (Magenta) in the epidermal cell of the root elongation zone under SCLIM. Scale bar = 1µm. See also Fig. 5e.

**Supplementary Movie 4
Description:** 4D movie of AP4M-GFP (Green) and CLC2-mKO (Magenta) in the epidermal cell of the root elongation zone under SCLIM. Scale bar = 1µm. See also Fig. 5g.

**Supplementary Movie 5
Description:** 4D movie of ST-iRFP (Cyan), GFP-SYP61 (Yellow), and CLC2-mKO (Magenta) in the epidermal cell of the root elongation zone under SCLIM. Scale bar = 1µm. See also Fig. 6.

**Supplementary Movie 6
Description:** 4D movie of GFP-SYP61 (Green) and CLC2-mKO (Magenta) in the epidermal cell of the root elongation zone under SCLIM. Scale bar = 1µm. See also Supplementary Fig. 4
