# Supplementary material for: Cargo sorting zones in the trans-Golgi network visualized by super-resolution confocal live imaging microscopy in plants
Source: Nat Commun. 2021 Mar 26;12:1901. doi: 10.1038/s41467-021-22267-0 (PMC7997971; doi:10.1038/s41467-021-22267-0)
Supplement: Supplementary file 10 — Reporting Summary [file 41467_2021_22267_MOESM10_ESM.pdf]

nature research

Corresponding author(s): Tomohiro Uemura
Akihiko Nakano

Last updated by author(s): Mar 4, 2021

Reporting Summary

Nature Research wishes to improve the reproducibility of the work that we publish. This form provides structure for consistency and transparency in reporting. For further information on Nature Research policies, see Authors & Referees and the Editorial Policy Checklist .

Statistics

For all statistical analyses, confirm that the following items are present in the figure legend, table legend, main text, or Methods section.

n/a	Confirmed	
☐	☒	The exact sample size (*n*) for each experimental group/condition, given as a discrete number and unit of measurement
☐	☒	A statement on whether measurements were taken from distinct samples or whether the same sample was measured repeatedly
☐	☒	The statistical test(s) used AND whether they are one- or two-sided<br>*Only common tests should be described solely by name; describe more complex techniques in the Methods section.*
☒	☐	A description of all covariates tested
☒	☐	A description of any assumptions or corrections, such as tests of normality and adjustment for multiple comparisons
☐	☒	A full description of the statistical parameters including central tendency (e.g. means) or other basic estimates (e.g. regression coefficient) AND variation (e.g. standard deviation) or associated estimates of uncertainty (e.g. confidence intervals)
☐	☒	For null hypothesis testing, the test statistic (e.g. *F*, *t*, *r*) with confidence intervals, effect sizes, degrees of freedom and *P* value noted<br>*Give P values as exact values whenever suitable.*
☒	☐	For Bayesian analysis, information on the choice of priors and Markov chain Monte Carlo settings
☒	☐	For hierarchical and complex designs, identification of the appropriate level for tests and full reporting of outcomes
☐	☒	Estimates of effect sizes (e.g. Cohen's *d*, Pearson's *r*), indicating how they were calculated

Our web collection on statistics for biologists contains articles on many of the points above.

Software and code

Policy information about availability of computer code

Data collection	SCLIM images were acquired using custom-made spinning disk confocal microscopy with custom-made software (Kurokawa et al., 2013; Kurokawa et al., 2019).<br>Conventional confocal laser scanning microscopy images were acquired using LSM780 with ZEN 2011 SP7 black v14.0.0.201 software (ZEISS).<br>Western blot images were captured using ImageQuant LAS 500 with ImageQuant TL v7.0 (GE Healthcare).
Data analysis	3D reconstruction, rendering, and quantification were carried out using Volocity v6.5 (Quorum Technologies).<br>Fluorescence intensity profiles of projection images were measured with ImageJ v1.51.<br>Statistical analysis was performed using R v3.3.1.

For manuscripts utilizing custom algorithms or software that are central to the research but not yet described in published literature, software must be made available to editors/reviewers. We strongly encourage code deposition in a community repository (e.g. GitHub). See the Nature Research guidelines for submitting code & software for further information.

Data

Policy information about availability of data

All manuscripts must include a data availability statement. This statement should provide the following information, where applicable:

- Accession codes, unique identifiers, or web links for publicly available datasets
- A list of figures that have associated raw data
- A description of any restrictions on data availability

The authors declare that all data supporting the findings of this study are available within the article and its supplementary information or are available from the corresponding authors on request.

Field-specific reporting

Please select the one below that is the best fit for your research. If you are not sure, read the appropriate sections before making your selection.

- [x] Life sciences
- [ ] Behavioural & social sciences
- [ ] Ecological, evolutionary & environmental sciences

For a reference copy of the document with all sections, see nature.com/documents/nr-reporting-summary-flat.pdf

Life sciences study design

All studies must disclose on these points even when the disclosure is negative.

Sample size	No statistical method was used to determine the sample size. The sample sizes were determined based on our experience. All experiments were performed at least in 3 independent biological replicates.
Data exclusions	No data were excluded from the analysis.
Replication	Experiments were repeated independently at least 3 times with reproducible results.
Randomization	Not relevant. The samples were analyzed in the same manner.
Blinding	Blinding was not applied to the data collection since the experiments were performed without prior knowledge of the outcome. The analyses of images were blinded. Key results were observed by more than 2 investigators.

Reporting for specific materials, systems and methods

We require information from authors about some types of materials, experimental systems and methods used in many studies. Here, indicate whether each material, system or method listed is relevant to your study. If you are not sure if a list item applies to your research, read the appropriate section before selecting a response.

Materials & experimental systems

n/a	Involved in the study	
[ ]	[x]	Antibodies
[x]	[ ]	Eukaryotic cell lines
[x]	[ ]	Palaeontology
[x]	[ ]	Animals and other organisms
[x]	[ ]	Human research participants
[x]	[ ]	Clinical data

Methods

n/a	Involved in the study	
[x]	[ ]	ChIP-seq
[x]	[ ]	Flow cytometry
[x]	[ ]	MRI-based neuroimaging

Antibodies

Antibodies used	Mouse anti-GFP antibody (Clontech, No. 632375; 1:20,000)<br>Rabbit anti-CHC antibody (Agrisera, AS10 690; 1:2,000)<br>Anti-Mouse IgG, HRP-Linked Whole Ab Sheep secondary antibody (GE Healthcare, NA931; 1:5,000)<br>Anti-Rabbit IgG, HRP-Linked Whole Ab Donkey secondary antibody (GE Healthcare, NA934; 1:5,000)<br>AlexaFluor 594-conjugated donkey anti-rabbit IgG secondary antibody (Jackson Immunoresearch, 711-585-152; 1:300)
Validation	All antibodies used are commercial and validated by the manufacturers.<br>·Mouse anti-GFP antibody; relevant website: https://www.takarabio.com/products/antibodies-and-elisa/fluorescent-protein-antibodies/green-fluorescent-protein-antibodies?catalog=632375<br>·Rabbit anti-CHC antibody; relevant website: https://www.agrisera.com/en/artiklar/clathrin-heavy-chain.html<br>·Anti-Mouse IgG, HRP-Linked Whole Ab Sheep secondary antibody; relevant website: https://www.gelifesciences.com/en/us/shop/protein-analysis/blotting-and-detection/blotting-standards-and-reagents/amersham-ecl-hrp-conjugated-antibodies-p-06260<br>·Anti-Rabbit IgG, HRP-Linked Whole Ab Donkey secondary antibody; relevant website: https://www.gelifesciences.com/en/us/shop/protein-analysis/blotting-and-detection/blotting-standards-and-reagents/amersham-ecl-hrp-conjugated-antibodies-p-06260<br>·AlexaFluor 594-conjugated donkey anti-rabbit IgG secondary antibody; relevant website: https://www.jacksonimmuno.com/catalog/products/711-585-152
